# Work-related quality of life of occupational therapists in Ireland

**DOI:** 10.1177/03080226231208055

**Published:** 2023-11-25

**Authors:** Victoria Hogan, Sinéad Hynes, Michael Hogan, Margaret Hodgins

**Affiliations:** 1 Discipline of Health Promotion, School of Health Sciences, University of Galway, Galway, Ireland; 2 Discipline of Occupational Therapy, School of Health Sciences, University of Galway, Galway, Ireland; 3 School of Psychology, University of Galway, Galway, Ireland

**Keywords:** Quality of working life, turnover intention, well-being, Irish occupational therapists, organisational constraints, workload

## Abstract

**Introduction:**

Research focused on workforce issues and the working conditions of occupational therapists in Ireland is limited. The aim of this study was to characterise quality of working life and well-being in Irish occupational therapists.

**Method:**

A cross-sectional, electronic survey of occupational therapists working in Ireland was conducted. The questionnaire included measures of quality of working life, well-being, workload, organisational constraints and turnover intentions.

**Results:**

A total of 157 occupational therapists completed the survey. Quality of work life and well-being scores were lower than available norms. Organisational constraints and workload predicted lower quality of working life, *F*(7,119) = 13.669, *p* < 0.0005, while organisational constraints was the only significant predictor of well-being, *F*(10,123) = 3.698, *p* < 0.0005. Lower quality of working life predicted turnover intention, *F*(1,139) = 63.004. *p* < 0.0005.

**Conclusion:**

Results indicate that organisational constraints and workload are significant predictors of lower quality of working life. Lower quality of working life is also related to turnover intention. Quality of working life studies such as this can provide a form of problem diagnosis, in highlighting organisational factors that impede quality of working life.

## Introduction

Recruitment and retention of occupational therapists internationally is an ongoing challenge (Morley et al., 2017; Nelson et al., 2015). Although very little empirical research on occupational therapy workforce issues in Ireland has been conducted, concerns have been raised by trade unions regarding retention in the face of structural change in the Irish health care system (Fórsa, 2018). This concern has intensified with the impact of COVID-19 on staffing levels. The need to address retention of health care professionals is critical in light of current personnel shortages and projected population growth, with failure to meet requisite staff increases reported as particularly high for occupational therapists (Keegan et al., 2022).

Stress, burnout and low job satisfaction are significantly associated with turnover intention in occupational therapists (Mertala et al., 2022; Park, 2021; Scanlan and Still, 2013). Research also shows that higher Quality of Working Life (QoWL) is associated with higher job satisfaction in general working populations (Sirghy et al., 2001; Wallace et al., 2007) and in occupational therapists (Rostami et al., 2021). Although QoWL is variously defined, there is agreement that it is a multidimensional construct (Easton and Van Laar, 2018; Wallace et al., 2007). QoWL is measured through a range of indicators and affords more useful information than unidimensional variables such as job satisfaction. This information is useful when seeking to understand potential ways of improving the working environment and service delivery.

Few studies have directly examined QoWL within the Irish Health Care setting, or with Irish occupational therapists. In the context of significant organisational change, service disturbance during the COVID-19 pandemic, and concerns about retention, the aim of this study is to explore QoWL and related constructs among Irish occupational therapists in order to characterise the workforce experience. Of particular interest is the relative contribution of personal or work-related variables influencing QoWL and whether QoWL influences turnover intention.

## Literature review

There is no consensus in the literature on an exact definition of QoWL (Sirghy et al., 2001). Reviews of the construct (e.g. Easton and Van Laar, 2018; Wallace et al., 2007), together identify 33 different possible components, although some overlap is evident. Many approaches divide components into personal and work-related, the latter being amenable to system or service-level intervention. For health care professionals, Easton and Van Laar (2018) employ a model and measure of QoWL comprised of six factors: (1) General Well-Being (GWB), (2) Home–Work Interface (HWI), (3) Job and Career Satisfaction (JCS), (4) Control at Work (CAW), (5) Working Conditions (WCS) and (6) Stress at Work (SAW). The model informed the development of a measure for Healthcare personnel (HCPs), which has been used in the NHS for benchmarking for service improvement (Easton and Van Laar, 2018).

QoWL has been examined widely in the healthcare setting. Employing Easton and Van Laar’s measure, McFadden et al. (2020) found half of a sample of 3290 United Kingdom (UK) health and social care workers experienced high QoWL and just over one-third (34.6%) experienced low QoWL. Variations in the measurement notwithstanding, there is evidence that QoWL is associated with turnover intention in many studies, including primary and tertiary care nurses (Kaddourah et al., 2018; Poku et al., 2022) and hospital employees (Shukla et al., 2017). There are few studies that measure QoWL directly in occupational therapists. A study of Iranian occupational therapists found moderate levels of QoWL, and a significant relationship between QoWL and job satisfaction (Rostami et al., 2021). Low scores on work-related factors were associated with low job satisfaction in a sample of Turkish occupational therapists (Abaoğlu et al., 2021). Rewards such as recognition and prestige, and both work/life balance and elements of job satisfaction were also shown to predict turnover intention in Australian occupational therapists (Scanlan and Still, 2013). Davey et al. (2014) report that low QoWL among occupational therapists is related to organisational factors, such as time limitations and excessive administrative paperwork. Abu Tariah et al. (2011) further reported that limited availability of treatment and assessment tools, overlooked areas of practice (e.g. home-based occupational therapy), and poor work benefits are linked to lower QoWL in occupational therapists. Additionally, adequate building space for the delivery of occupational therapy affected job satisfaction (Abu Tariah et al., 2011). No firm conclusions can be made from the available QoWL literature in occupational therapists and it is important to recognise potential differences across countries and regions in service provision, training and level of development of the profession (World Federation of Occupational Therapists (WFOT), 2020), which may influence perceptions of QoWL. Grote and Guest (2017) have called for a renewed focus on QoWL as a means to address the challenges to worker well-being inherent across workplaces today.

## Occupational therapy in Ireland

In 2019, there were 2700 practising occupational therapists in Ireland, which approximates to six therapists per 10,000 population. Of these 35% (*n* = 938) were registered with the Association of Occupational Therapy Ireland (AOTI) (WFOT, 2020). Occupational therapists in Ireland can work in public or private practice, with some working part-time across both. The majority are employed through the statutory health service (Health Service Executive (HSE)) and work in acute hospital services or within community healthcare organisations (CHO) across the country. In the face of known staff shortages and retention issues (Fórsa, 2018) the occupational therapy workforce in the public (acute) hospital sector saw growth of 3.8% on average yearly between 2015 and 2019 (Keegan et al., 2022). Going forward, particularly large increases (2.7–3.3% annually) are projected as needed in the acute hospital sector, based on expected increases in older age groups requiring hospital-based treatment. This is in addition to turnover rates which are reported to be in the region of 8% for therapy grades, a higher level of churn in the health care services than any other group with the exception of medical consultants (Fórsa, 2018). Specifically, between 183 and 214 additional occupational therapists are required by 2035 (Keegan et al., 2022).

Over the last ten years, occupational therapists working in the HSE have worked through significant change and disruption. The introduction of CHO in 2013 brought about significant organisational change within the HSE along with a restructuring of teams. The CHOs deliver primary care services, including General Practitioner services, public health nursing, physiotherapy, speech and language therapy and occupational therapy. There is a revised focus on integrated care and a shift in leadership and management. In 2017, ‘Sláintecare’ was introduced, a large-scale reform programme aiming to deliver a single-tier integrated universal health care system based on medical need in the place of two-tier public-private mix, characterised by inbuilt inequalities in access, long waiting lists and staff shortages. However, implementation has been slow, with early milestones missed and altered (Burke et al., 2018). Implementation was further challenged with the onset of COVID-19.

As with many other countries, the COVID-19 pandemic brought unprecedented challenges to the health care system in Ireland and cut across planned organisational changes and proposed increases in staffing levels. Not only did significant spending have to be diverted, many front-line staff were redeployed to tracing and vaccination services. In April 2020, 3555 community health care staff were redeployed. This dropped to 815 by November 2020, 42 of which were occupational therapists.^1^ During COVID year 2020, the total number of patients seen in community teams was 22% below the target for the year, with an increase of 14% in patients awaiting an assessment (National Public Health Emergency Team, 2020). The impact of these structural changes and upheavals in service delivery and staffing on the well-being of occupational therapists and relatedly retention is not well researched in the Irish setting.

There is limited research on Irish occupational therapy workforce issues using QoWL measures, although concerns have been raised about the complex and cumbersome referral procedures from acute to community care (Houston, 2019), lack of resources, inconsistencies and poor access to technology during the COVID-19 pandemic (Culleton, 2021), in addition to dissatisfaction with HSE recruitment procedures (AOTI, 2020). With regard to changes in working conditions, a survey of 403 Irish occupational therapists working across all areas, found that two-thirds feel more negative about their job over the past 12 months, 55% have considered leaving their job, and 44% have considered leaving the profession (AOTI, 2022). This level of turnover intention is a very significant increase from the figure of 14% recorded in the survey on recruitment issues, two years previously, indicating seriously deteriorating working conditions (ibid). Staff shortages were a particular concern with 81% believing the service to be understaffed, 66% reporting increased service demand and 46% reporting increased targets or service expectations.

In this context, further exploration of the working life of Irish occupational therapists warrants urgent attention, including factors affecting the mental health and well-being of therapists, and the real potential for a nosedive in retention with an attendant impact on the provision of sustained, high-quality services that help children and adults to participate in everyday meaningful activities (AOTI, 2022). To this end, a cross-sectional study was conducted, in which the main variables of interest were QoWL, well-being, and turnover intention. Given the particular context in Ireland of staff shortages and redeployment, workload and organisational constraints were also measured.

## Method

A cross-sectional, quantitative, multi-variate study design was employed for this study. All participants provided their informed consent before completion of the survey. Ethical approval was granted by the University of Galway, Research Ethics Committee in March 2022.

### Data collection

Occupational therapists registered with the AOTI in 2022 (*N* = 1250) were invited to participate in the study. An email invitation containing the study information sheet and link to the electronic survey in Microsoft Forms was distributed by the AOTI to members. A reminder email was subsequently circulated by the AOTI, and social media, that is, Twitter was also used to advertise the study and distribute the questionnaire to occupational therapists, as only 35% of occupational therapists in Ireland are members of AOTI, according to WFOT. A total of 157 responses were returned, giving an approximate response rate of 12% of the AOTI registered workforce, and (employing the WFOT figure of 2700) 6% of the total workforce.

### Measures

The questionnaire was composed of five measures: (1) QoWL Scale (Van Laar et al., 2007), (2) WHO Five Well-Being Index (WHO-5) Wellbeing Index (World Health Organization (WHO), 1998), (3) Turnover Intentions Scale (Michaels and Spector, 1982), (4) Quantitative Workload Inventory (Spector and Jex, 1998), and (5) Organisational Constraints Scale (OCS) (Spector and Jex, 1998). Internal consistency (Cronbach’s alpha) statistics for most measures exceeded 0.7 (see Table 3). Calculation of Cronbach alpha for the Organisational Constraints scale is not recommended as it is used to generate an aggregate index of constraints and items included do not all measure the same underlying construct (Spector and Jex, 1998). One open-ended question was also included, which asked respondents ‘Is there anything else that you would like to add about your quality of working life?’ Further detail on the quantitative measures is presented below.

#### Demographic and work questions

Demographic questions included age, gender, ethnicity, disability status, marital status and caring responsibilities. Work questions investigated professional area of work, job tenure and role, time of gaining professional qualification, hours of work, additional working hours/overtime.

#### QoWL Scale

Based on McFadden et al.’s (2020) study on HCPs in the UK, the QoWL Scale (Van Laar et al., 2007) was employed. The QoWL Scale includes six factors: (1) GWB (six items), (2) HWI (three items), (3) JCS (six items), (4) CAW (three items), (5) WCS (three items), and (6) SAW (two items). The items are answered on a five-point Likert-type scale ranging from ‘Strongly Disagree’ to ‘Strongly Agree’. Factor scores and an overall score were computed as per Easton and Van Laar’s (2018) scoring instructions. The average scores were compared to the UK normative data, derived from studies in the NHS (Easton and Van Laar, 2018). The measure is psychometrically strong and has been used in numerous studies (Easton and Van Laar, 2018).

#### WHO Five Well-Being Index

Well-being was measured using the WHO-5 (WHO, 1998). The measure includes five items (e.g. ‘I have felt cheerful and in good spirits’), each of which are rated by respondents using a five-point Likert-type scale ranging from 0 (*at no time*), to 5 (*all of the time*). Total raw scores (ranging from 0 to 25) are multiplied by 4 to provide a final scale score, which ranges from 0 to 100 (Topp et al., 2015). Scores less than 50 indicate poorer well-being, and a WHO-5 cut-off score of ⩽50 is recommended when screening for clinical depression (Topp et al., 2015). The WHO-5 is widely used and is psychometrically sound (Topp et al., 2015).

#### Turnover Intention Scale

The Michaels and Spector (1982) three-item scale was used to measure turnover intention. Items are scored on a six-point Likert-type scale ranging from ‘strongly disagree’ to ‘strongly agree’. An example item is ‘I often seriously consider leaving my current job’. Higher scores indicate greater turnover intentions. The Turnover Intention Scale (TIS) has demonstrated predictive validity (Michaels and Spector, 1982).

#### Quantitative Workload Inventory and OCS

These measures were selected as they are often used together, and norms are available (Spector and Jex, 1998). The Quantitative Workload Inventory (QWI) includes five items and has demonstrated good internal consistency (i.e. 0.82 across 15 studies, Spector and Jex, 1998). Participants report how often different situations occur (e.g., ‘how often does your job require you to work very fast?’). A five-point Likert-type scale ranging from ‘less than once per month’ to ‘several times per day’ is used for responses. Higher scores indicate higher workload (Spector and Jex, 1998). The OCS includes 11 items that measure how difficult respondents find it to do their job because of constraints (e.g. ‘Poor equipment or supplies’). A five-point Likert-type scale ranging from ‘less than once per month’ to ‘several times per day’ is used for responses. Higher scores indicate higher organisational constraints (Spector and Jex, 1998).

### Data analysis

All descriptive and inferential statistics were conducted using SPSS, Version 26. Means and standard deviations (SDs) were calculated for continuous variables, and frequencies and percentages were calculated for categorical level data. A series of regression analysis were conducted to address the study objectives. Data derived from the open-ended question was analysed using content analysis.

## Results

Table 1 presents the demographic profile of the sample. Ninety-four percent of survey respondents (*n* = 148) were female, 5% (*n* = 8) were male, and 1% (*n* = 1) preferred not to say. The majority were aged between 30 and 49 years, married, and working full time in the public sector. A larger proportion of participants did not have children (59%), and just under half had completed postgraduate qualifications (48%).

**Table 1.** Sample characteristics.

Variable	*N* = 157	%
Age		
18–29	27	17
30–39	48	31
40–49	43	27
50–59	30	19
60–65	8	5
65+	1	1
Marital status		
Single	43	28
Married	90	57
Co-habiting	16	10
Divorced/separated	5	5
Children		
Yes	64	41
No	92	59
Qualification		
Postgraduate	76	48
Undergraduate	80	52
Employment status		
Full time	126	81
Part time	30	19
Sector		
Public	122	78
Private	17	11
Voluntary/community/other	17	11
Years of work experience		
<5	30	19
6–10	19	12
11–20	52	33
21–30	40	26
>30	16	10

**Figure 1.**
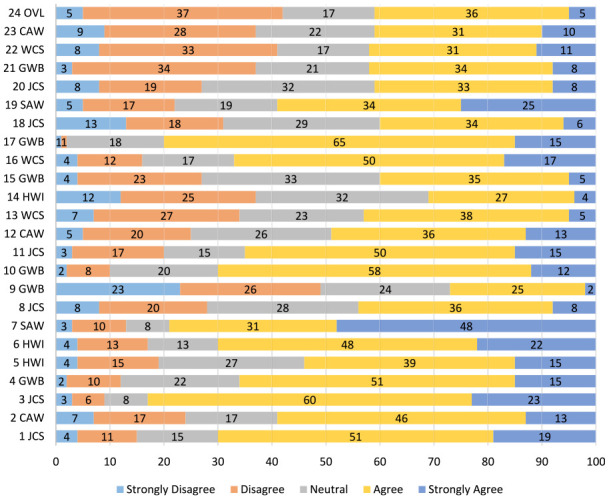
Distribution of responses to the items on the WrQoL questionnaire presented in percentages. OVL: Quality of Work-life Overall; CAW: Control at Work; WCS: Working Conditions; GWB: General Well-being; JCS: Job Career Satisfaction; SAW: Stress at Work; HWI: Home Work Interference ; WrQoL: Work-related quality of life.

Figure 1 indicates the distribution of the responses to the QoWL questionnaire items, presented as percentages. Regarding satisfaction with overall QoWL (24OVL), roughly equal proportions of participants were satisfied (41%) and dissatisfied (42%) with their working lives. Responses to the six GWB items indicate a mixed picture, with 60% reporting that they were satisfied with their lives (10GWB) and 26% reporting feeling unhappy and depressed (9GWB). Current levels of happiness (21GWB) was also mixed with 42% agreeing that recently they were feeling reasonably happy, while 37% disagreed. Work–life balance was reported as broadly positive across the three HWI items, with 70% agreeing/strongly agreeing that their work hours/patterns suit their personal circumstances (6HWI), while 54% agreed/strongly agreed that their employers provide adequate facilities and flexibility to support work–life balance (5HWI). Responses to 14HWI regarding the promotion of work–life balance by line managers were mixed. A high proportion agreed/strongly agreed that their work situation was safe (16WCS, 67%); however, only 43% agreed that work conditions were satisfactory (22WCS) and only 42% agreed that they are provided with the necessary work-related resources to work effectively by management (13WCS). Some aspects of job and career satisfaction were very positive, with high proportions agreeing/strongly agreeing that they had clear goals and aims at work (1JCS, 70%), could use their abilities at work (3JCS, 83%) and were encouraged to develop new skills (11JCS, 65%). Levels of agreement were lower in response to items regarding satisfaction with training (20JCS, 41%), career opportunities (18JCS, 40%) and receiving acknowledgement for good work (8JCS, 44%). In response to the control at work questions, 59% agreed/strongly agreed that they could voice their opinions and influence changes at work (2CAW), that they were involved in decisions that affected them at work (12CAW, 49%) and to a lesser extent the public (23CAW, 41%). The majority (79%) agreed/strongly agreed that they often felt under pressure at work (7SAW), while 59% reported experiencing excessive levels of stress (19SAW).

Table 2 presents the means and SDs for all questionnaire measures. Scale scores were compared to published norms for the QoWL measure, the QWI measure and the OCS measure. All QoWL factor scores and the overall scale score in this study were lower than the published norms. For example, the average overall score of 3.16 equates to the 30th percentile of a sample of NHS study participants and indicates that the average score in this study is equal or higher than 30% of NHS healthcare workers. The average score (18.8) on the QWI was also higher than the published norm (16.5), as was the OCS score (25.35; norm, 21.3). The average WHO-5 score was 53.5, is slightly above the cut-off score of 50 used to denote low well-being (Topp et al. 2015). In total, 43% of the respondents had scores below the cut-off score of 50. Descriptive statistics also indicate that 57% of participants had considered resigning from their positions, with 46% having started to look for other jobs. However, only 33% agreed that they intended to quit their position.

**Table 2.** Means, SDs and Cronbach’s alphas on questionnaire measures.

	Mean	SD	Normative data comparison	Cronbach’s alpha
WrQoL	3.16	0.58	30th percentile	
1. General Well-being Factor	3.49	0.65	30th to 40th percentile	0.773
2. Home–Work Interface	3.25	0.90	30th to 40th percentile	0.808
3. Job Career Satisfaction	3.41	0.73	Close to 40th percentile	0.794
4. Control at Work	3.25	0.81	Close to 40th percentile	0.524
5. Stress at Work	2.15	1.05	30th to 40th percentile	Not calculated – only two items in scale
6. Working Conditions	3.26	0.76	Close to 40th percentile	0.494
Turnover intention	10.22	2.35		0.812
WHO-5	53.54	18.37		0.868
Organisational constraints	25.35	9.31	Higher than norm	
Quantitative workload inventory	18.80	5.23	Higher than norm	0.907

SD: standard deviation; WHO-5: WHO Five Well-Being Index; WrQoL: Work-related quality of life.

### Regression analyses

Stepwise multiple regression was employed to determine the influence of personal and work-related variables on QoWL. Because the QoWL composite score considers general well-being, the WHO-5 well-being measure was not included as a potential predictor variable to avoid problems with multicollinearity. Among the predictor variables employed in the regression analysis, Pearson’s correlation analysis indicated that only a small number were correlated. Most correlations were within the moderate range. For example, a positive correlation was observed between QWI scores and the OCS scores, *r* = 0.572, *p* < 0.001. Variance inflation factors (VIF) for all predictor variables were checked and they ranged from 1.085 to 2.230, indicating that multicollinearity among the predictor variables was not a significant issue.

Variables were entered in three blocks, first the personal variables were entered (age, marital status, children) followed by work-related variables (job permanence, full-time/part-time status, years’ work experience, sector, hours per week), and finally the QWI and OCS variables. A significant model was produced, *F*(7,119) = 13.669, *p* < 0.0005, that explained 41.3% of the variance (adjusted *R*^2^ = 0.413). Table 3 presents information for the predictor variables included in the final model.

**Table 3.** The unstandardised and standardised regression coefficients for the variables included in the model.

Variable	*B*	SE *B*	β	Significance
Full time/part time	0.257	0.148	0.176	0.086
Job permanence	0.049	0.043	0.086	0.256
Work experience	−0.047	0.031	−0.109	0.127
Sector	−0.028	0.047	−0.043	0.554
Work hours	0.157	0.092	0.173	0.092
Organisational Constraints	−0.027	0.005	−0.483	0.000
Quantitative workload	−0.029	0.009	−0.269	0.002

SE: standard error.

A second stepwise multiple regression analysis was run examining the influence of the same predictor variables on mental well-being as measured by the WHO-5. A significant model was produced, *F*(10,123) = 3.698, *p* < 0.0005, accounting for 16.9% of the variance (adjusted *R*^2^ = 0.169). Organisational Constraints was the only significant predictor variable, *B* = −0.186, *B* standard error (SE) = 0.05, β = −0.376.

Linear regression was conducted to determine whether QoWL influenced turnover intention. Using the enter method, a significant model was produced: *F*(1,139) = 63.004, *p* < 0.0005. The model explained 30.7% of the variance (adjusted *R*^2^ = 0.307), *B* = −2.251, *B* SE = 0.284, β = −0.558.

### Open question

Sixty-eight of the 157 respondents offered additional comments when asked ‘Is there anything else that you would like to add about your quality of working life?’. The majority of comments were negative, referring predominantly to workload issues: the words ‘demand’, ‘stress’ and ‘pressure’ occurring repeatedly. These comments were principally linked to issues such as poor clinical governance, a poorly planned and disorganised service and serious staffing issues, but also limited career opportunities and childcare supports influencing working life quality. Respondents were concerned about high patient to therapist ratios and its impact on workload. There were also concerns expressed about the quality of intervention, which is troubling from a professional perspective. There were several references to plans to leave working within a statutory service and moving into private practice; indeed some respondents had already made this move. The small number of positive comments were in the context of the autonomy and control associated with being self-employed. On balance, the comments from this subset of respondents indicate serious problems meeting professional responsibilities in the context of an under-resourced and over-bureaucratised service.

## Discussion and implications

The current study examined levels of QoWL in a sample of Irish Occupational Therapists and the influence of personal variables (age, marital status, children) and work-related variables (job permanence, full-time/part-time status, years’ work experience, sector, hours per week, workload and organisational constraints) on their QoWL. Overall, QoWL levels reported by participants in the current study were worryingly low, and specific work-related variables (i.e. workload and organisational constraints), but not personal variables, accounted for significant variance in the QoWL scores observed. Lower QoWL scores in turn predicted higher turnover intentions.

Notably, less than half of participants in the current study (41%) report satisfaction overall with their QoWL, with just as many being dissatisfied (42%). These satisfaction ratings are low compared with those of other published studies (Easton and Van Laar, 2018; McFadden et al., 2020) and are reinforced by the negative tone of many comments in response to the open-ended question. The overall QoWL score and the six factor scores in this study were at the lower end of the distribution compared to the NHS norms for HCPs (Easton and Van Laar, 2018). While the NHS norms (Easton and Van Laar, 2018) relate to pre-COVID QoWL, the low QoWL scores in this study also compare unfavourably with McFadden et al.’s (2020) finding of 50% HCPs reporting high QoWL, measured during COVID.

High organisational constraints and high workload were key predictors of low QoWL in the current study. it is notable that the OCS mean score for participants in the current study (25.35) was higher than the available norm (21.3), indicating high levels of organisational constraints. These findings are consistent with specific organisational problems previously identified in the Irish context (Culleton, 2021; Houston, 2019). The OCS has been characterised as a causal indicator scale that combines a range of factors that potentially interfere with one’s work and result in increased strain, lower well-being, counterproductive work behaviour and physical symptoms (Pindek and Spector, 2016). In addition to its impact on QoWL, the findings of this study are consistent with that of Pindek and Spector (2016) as higher OCS also predicted lower well-being in this study. At the same time, further research is needed to clarify the precise impact of specific organisational constraints on QoWL and well-being in Occupational Therapists. A mixed-methods approach to future research would be valuable to further understand quantitative relationships between key variables across different working contexts, and the qualitative nature of specific organisational constraints on the QoWL.

In addition to organisational constraints, regression analysis also revealed that high workload was a significant predictor of QoWL. Heavy workloads were evident as the QWI mean score in the current study (18.8) was higher than the available norm (16.5). The findings of the current study indicating both workload and organisational constraints as significant predictors of QoWL are interesting in light of Pindek and Spector’s finding that organisational constraints are more noticeable when workloads are heavy (Pindek and Spector, 2016). It may be the case that high workload amongst occupational therapists in Ireland exacerbates the negative influence of organisational constraints on QoWL. Frustration in relation to workload was also explicitly mentioned by a number of participants in the current study in response to the open-ended question.

In relation to wellbeing, the average score in this study of 53.5 is similar to the average score of 52 reported in the GreenCovid survey conducted in Ireland (Guzman et al., 2020). However, 43% of the occupational therapists in this study had scores <50, which is the cut-off level denoting low well-being. The majority of respondents (79%) reported that they often felt being under pressure at work and 59% reported experiencing excessive levels of work-related stress, considerably higher than the 37% experiencing burnout in the AOTI survey (AOTI, 2022).

Turnover intentions among the sample were high, with 46% reporting that they were looking for other jobs, while a third reported intending to leave their job. These findings are consistent with the critical issue of turnover identified in the AOTI survey (AOTI, 2022). As noted, QoWL predicted turnover intention in the current study, underscoring the importance of addressing organisational factors in efforts to retain staff working in the sector. Turnover and intentions to seek out alternative working arrangements were also commonly noted in the responses to the open-ended question. Acknowledging that the responses to the open-ended question only reflect a sub-set of the overall respondents, nevertheless, the working environment described by study participants can be considered very challenging. In efforts to achieve a better QoWL, one strategy noted by participants is to move from the public to private sector. However, if this strategy dominates workforce trends, the drain of qualified personnel from the public sector will negatively affect patient services and further exacerbate conditions for those who remain (Poon et al., 2022). Poon et al. (2022) note in their meta-analysis that many of the factors associated with turnover intention were known factors such as work and organisational issues that were already problematic prior to the COVID-19 pandemic. Rostami et al. (2021) have previously found that QoWL is related to job satisfaction in occupational therapists. This study has added to the extant literature by demonstrating that lower QoWL also predicts higher turnover intention.

Poon et al. (2022) recommend further examination of working conditions as a means of generating advice for policy makers regarding retention of healthcare workers. Therefore, the current study is timely as it provides an opportunity to reflect on features of working life for Irish occupational therapists following the acute stage of the pandemic. Our study findings have implications for both policy and practice. For example, the ambitious plans to increase occupational therapy student numbers in Ireland to address staff shortages and projected future needs is an important policy decision nationally. However, in practice, graduates will face a challenging work environment if stressors such as workload and organisational constraints identified in this study are not addressed. Resources must be provided to steadily improve QoWL. Based on the current study, key areas of focus include the provision of necessary work-related resources by management, enhancement of training provision and viable career pathways. In addition, greater involvement in decision-making and efforts to reduce pressure at work are required, as current work systems are a source of stress and distress.

The findings of the current study also highlight the need to engage in a comprehensive international analysis of the working conditions for occupational therapists in different countries since the onset of the COVID-19 pandemic, coupled with efforts to share international best practice and design solutions that optimise work conditions and patient care. While the results of the current study compare unfavourably with the findings in relation to QoWL in other countries, it is clear that there is pressure on health services across the world currently related to economic pressures, changing demographics, and changing patterns of ill health and disability across nations (Szabo et al., 2020). Continued efforts by WFOT are required to reinforce international cooperation in the occupational therapy sector such that the profession can flourish into the future and support effective service delivery.

The objective of this study was to characterise QoWL among the occupational therapy community in Ireland, given that there is little known about key elements of QoWL for this professional group. One previous Irish study, which examined stress and burnout levels within the profession, heralded serious concerns about low morale and well-being and the potential impact on retention and service delivery (AOTI, 2022). This study is timely given on-going understaffing issues, the impacts of the COVID-19 pandemic on service provision, and ongoing changes within the Irish public healthcare service. There is a clear need to increase numbers within the profession over time in order to support healthcare needs nationally into the future, but the current study also highlights the need to reflect carefully on how best to redesign working environments to enhance working conditions.

## Study limitations

A number of study limitations are evident. The response rate (6%) was low and may give rise to concerns in relation to self-selection. However, the response rate is comparable to other QoWL studies such as Zubair et al. (2017). Notwithstanding the low response rate, the sample is considered reflective of the Irish occupational therapy community, which is female dominated (WFOT, 2020). Given the larger proportion of respondents from the public sector, our findings may not be representative of QoWL issues in the private sector. The responses to the open-ended questions represent only a sub-set of the overall sample and therefore may not be representative of the opinions of the sample overall.

## Conclusion

In the face of challenging and possibly deteriorating work conditions, there is a clear need to address QoWL for Irish occupational therapists. The findings of this study indicate that workload and organisational constraints are particularly challenging and highlight the potential for a drift to the private sector, which will have implications for services for disadvantaged and marginalised populations. Further research could include a larger scale survey with sufficient resources to secure a nationally representative sample, alongside a companion qualitative investigation to provide additional detail on working conditions.

**Key findings**

- Quantitative workload and organisational constraints were the strongest predictors of QoWL in Irish occupational therapists.
- Organisational constraints was also the only significant predictor of well-being.
- Lower QoWL predicted turnover intention in Irish occupational therapists.

**What the study has added?**

This study provides the first analysis of work factors that may positively or negatively affect working experience, well-being and turnover intention among Irish occupational therapists and indicates areas for improvement.

## Notes

1. No later redeployment figures available.
